# An Adaptive Neural Mechanism for Acoustic Motion Perception with Varying Sparsity

**DOI:** 10.3389/fnbot.2017.00011

**Published:** 2017-03-09

**Authors:** Danish Shaikh, Poramate Manoonpong

**Affiliations:** Embodied AI and Neurorobotics Laboratory, Centre for BioRobotics, Maersk Mc-Kinney Moeller Institute, University of Southern DenmarkOdense, Denmark

**Keywords:** acoustic motion perception, binaural acoustic tracking, sound localisation, correlation-based learning, lizard peripheral auditory system

## Abstract

Biological motion-sensitive neural circuits are quite adept in perceiving the relative motion of a relevant stimulus. Motion perception is a fundamental ability in neural sensory processing and crucial in target tracking tasks. Tracking a stimulus entails the ability to perceive its motion, i.e., extracting information about its direction and velocity. Here we focus on auditory motion perception of sound stimuli, which is poorly understood as compared to its visual counterpart. In earlier work we have developed a bio-inspired neural learning mechanism for acoustic motion perception. The mechanism extracts directional information via a model of the peripheral auditory system of lizards. The mechanism uses only this directional information obtained via specific motor behaviour to learn the angular velocity of unoccluded sound stimuli in motion. In nature however the stimulus being tracked may be occluded by artefacts in the environment, such as an escaping prey momentarily disappearing behind a cover of trees. This article extends the earlier work by presenting a comparative investigation of auditory motion perception for unoccluded and occluded tonal sound stimuli with a frequency of 2.2 kHz in both simulation and practice. Three instances of each stimulus are employed, differing in their movement velocities–0.5°/time step, 1.0°/time step and 1.5°/time step. To validate the approach in practice, we implement the proposed neural mechanism on a wheeled mobile robot and evaluate its performance in auditory tracking.

## 1. Introduction

Historically motion perception has been extensively studied in the context of visual tracking. This comes as no surprise as it is the dominant sense for humans and most animal species. In humans it plays an important role in visuomotor coordination tasks such as catching a ball (Oudejans et al., 1996). In the animal kingdom, motion perception is a crucial element that is relevant to sustenance and survival. It is particularly important in conditions where the target being tracked is sporadically occluded (Morgan and Turnbull, 1978) such as a predator tracking a moving prey that occasionally disappears from view behind trees.

A simple correlation-based neural circuit for motion detection in vision that selectively responds to direction and velocity given monocular visual input has been proposed decades ago by Reichardt (1969). Such low-level motion detectors however have not been reported for audition. Auditory motion perception has therefore been suggested by Carlile and Leung (2016) to exist as a higher level system, similar to binocular, attention-modulated third-order visual motion detectors. The authors furthermore suggest that such third-order systems likely respond to snapshots of location information extracted from binaural cues. However, visual tracking experiments in the context of smooth eye pursuit that utilised periodic occlusion of the target indicate that target velocity may be a significant spatial information source (Barnes and Asselman, 1992; Churchland et al., 2003; Orban de Xivry et al., 2008).

Given a means to estimate a moving target's relative location and information regarding the time during which subsequent estimates are determined, the target's velocity can be derived. Here we demonstrate that the target velocity for continuous unoccluded as well as occluded acoustic targets could be learned based on the determination of these two pieces of information. We frame the problem of acoustic motion perception as an active acoustic tracking task. Active acoustic tracking entails movement of the acoustic organs to track an object, which is a natural auditory tracking behaviour. The dynamics of auditory tracking in cats with disconnected optical nerves, which disabled visual processing, have been behaviourally investigated (Beitel, 1999). The recorded head motion of these animals while tracking a series of click sounds emitted by a rotating loudspeaker suggested sound localisation being performed in a series of steps. The animals first displayed a rapid saccade-like head-orienting response to localise the target within the frontal sound field. This was followed by successive head movement cycles where the head would overshoot and pause, ensuring that the target's location remained close to the median plane.

### 1.1. Auditory localisation cues for spatial motion perception

There are three types of cues available for auditory localisation–the difference in arrival times of a sound (interaural time difference or ITD), the difference in sound level (interaural level difference or ILD) and spectral information (direction-dependent energy minimisation over the entire frequency spectrum due to filtering by the outer ear). Several animals such as frogs, crickets and lizards utilise only ITD cues for sound localisation. For these animals spectral and ILD cues are unavailable due to lack of pinnae and the diffraction of sound around the head respectively. Using difference cues for localisation requires two ears with a frequency-dependent displacement between them. Generating ILD cues requires a sufficiently large head between the ears. The dimensions of the head should however be at least greater than the half-wavelength of the sound signal to successfully generate ILD cues. This creates an acoustic shadow inside which the relative sound amplitude is reduced. ITD cues can however be generated without the need of such obstructions, but do depend on the displacement between the ears and the angle of incidence of the sound with respect to the median plane. Here we restrict ourselves to acoustic tracking of a moving sound signal using only ITD cues extracted from microphones.

A sound signal moving in a given direction with a constant velocity with respect to the microphones generates dynamically varying ITD cues. The instantaneous values of these cues are dependent on the relative instantaneous position of the sound signal, while the rate with which they vary is dependent on the relative movement speed of the sound signal. Actively tracking a moving sound signal therefore requires transforming these relative position- and velocity-dependent cues into a desired behaviour, for example robotic orientation or phonotaxis. One must first determine the instantaneous spatial location of the sound signal to within the desired threshold of the instantaneous tracking error. This localisation must then be successively repeated sufficiently quickly to minimise the tracking error.

### 1.2. Relevance of acoustic motion perception

There are several applications where actively tracking an acoustic target can be of interest. In robot phonotaxis applications, the robot could localise acoustic signals and navigate toward them (Reeve and Webb, 2003; Oh et al., 2008). In audio-visual teleconferencing systems, dynamically-steered microphone systems that automatically orient toward a speaker as they move about in a room could maximise the power of the incoming audio signal or orient a video camera toward the current speaker (Wang and Chu, 1997; Brandstein and Ward, 2001). Social robots that respond to sound and/or speech input from the human are another example. The verbal human-robot interaction element in social robots is deemed to be more natural and richer if the robot's acoustomotor response orients and maintains its gaze as well as auditory focus on the subject of interest (Nakadai et al., 2000; Okuno et al., 2003) in motion. For example, a human walks around in a room while addressing the robot via either directed or undirected speech commands.

Conventional acoustic tracking techniques (Liang et al., 2008a; Tsuji and Suyama, 2009; Kwak, 2011; Ju et al., 2012, 2013; Nishie and Akagi, 2013) are passive in that they require no movement of the listener. All of these techniques extract ITD cues for localisation by utilising multi-microphone arrays with at least four microphones. Typical arrays comprise an order of magnitude more microphones arranged in various geometric configurations such as linear, square, circular or in distributed arrays. ITD-based sound localisation and tracking techniques also tend to utilise computationally intensive algorithms such as particle filtering to compute the relative sound signal location from raw ITD data (Ward et al., 2003; Lehmann, 2004; Valin et al., 2007; Liang et al., 2008b; Ning et al., 2015). More conventional approaches are based on the generalised cross-correlation technique (Knapp and Carter, 1976) or the more recent steered response power technique (DiBiase, 2000; DiBiase et al., 2001; Zotkin and Duraiswami, 2004; Dmochowski et al., 2007; Cai et al., 2010; Wan and Wu, 2010; Marti et al., 2013; Zhao et al., 2013; Lima et al., 2015). Employing a larger number of microphones can improve localisation accuracy but at the expense of greater computational complexity and costly hardware for synchronisation and processing of multi-channel acoustic signals.

### 1.3. Contribution of the present work

We have previously reported a system for acoustic motion perception (Shaikh and Manoonpong, 2016) employing two microphones that implements a neural learning mechanism. The learning utilises a mathematical model that mimics the functionality of the auditory processing performed by the lizard peripheral auditory system (Wever, 1978). The system provides sound direction information and has been characterised via bio-faithful mathematical modelling (Zhang, 2009). The parameters of the model have been determined from biophysical data recorded from live lizards (Christensen-Dalsgaard and Manley, 2005). The model has also been implemented on a number of robotic platforms as reviewed in Shaikh et al. (2016). The neural learning mechanism has been adapted from the Input Correlation (ICO) learning approach (Porr and Wörgötter, 2006), which itself has been derived from a class of differential Hebbian learning rules (Kosko, 1986). The neural mechanism is considered to be a first step toward the development of a biologically-plausible neural learning mechanism for acoustic motion perception. The mechanism has been validated in simulation for tracking a continuous unoccluded acoustic signal moving with a constant and unknown angular velocity along a semi-circular trajectory. It has also been shown to learn various target angular velocities in separate simulated trials.

Here we implement the neural learning mechanism and compare its tracking performance for three different types of sound signals–continuous unoccluded, periodically occluded and randomly occluded. We first implement the neural mechanism in simulation that allows a robotic agent to learn to track a virtually-moving continuous unoccluded sound signal for a set of three different and unknown target angular velocities. As earlier the virtual sound signal is a pure tone moving along a semi-circular trajectory. To validate the tracking performance in practice, the learned synaptic weights representing a given target angular velocity are then used directly on a wheeled mobile robot that also implements the neural mechanism.

Next we implement another instance of the neural mechanism in simulation to learn to track a periodically occluded acoustic signal, moving with a constant but unknown angular velocity along a semi-circular trajectory. The occluded acoustic signal is implemented as an intermittent signal, i.e., it has a continuous unoccluded sound for a constant interval followed by complete silence for a constant interval. The silence implies that the signal is occluded and therefore inaudible. The acoustic tracking performance is evaluated in simulation for a constant “duty cycle” of sound emission. In this manner, the acoustic tracking performance is again evaluated for a set of three different target angular velocities identical to those used earlier. An instance of the simulation results is validated in practice via robotic trials with the wheeled mobile robot.

Finally, we implement a third instance of the neural mechanism in simulation to learn to track an occluded acoustic signal as described earlier, however with a randomly varying duty cycle. The signal moves as before with a constant but unknown angular velocity along a semi-circular trajectory. We evaluate the acoustic tracking performance for a set of three different target angular velocities identical to those used earlier. The main contribution of this work lies in systematically investigating the comparative performance of a neural closed-loop learning mechanism in learning the angular velocity of an acoustic stimulus with varying sparsity.

This article is organised in the following manner. Section 2 provides background information about the lizard peripheral auditory system and its equivalent model as well as about ICO learning. Section 3 presents the adaptive neural acoustic tracking architecture, the experimental setup and the robot model. Section 4 shows the experimental results in both simulation and practice. Section 5 summarises the work and discusses future directions.

## 2. Background

### 2.1. The lizard peripheral auditory system

**Figure 1:**
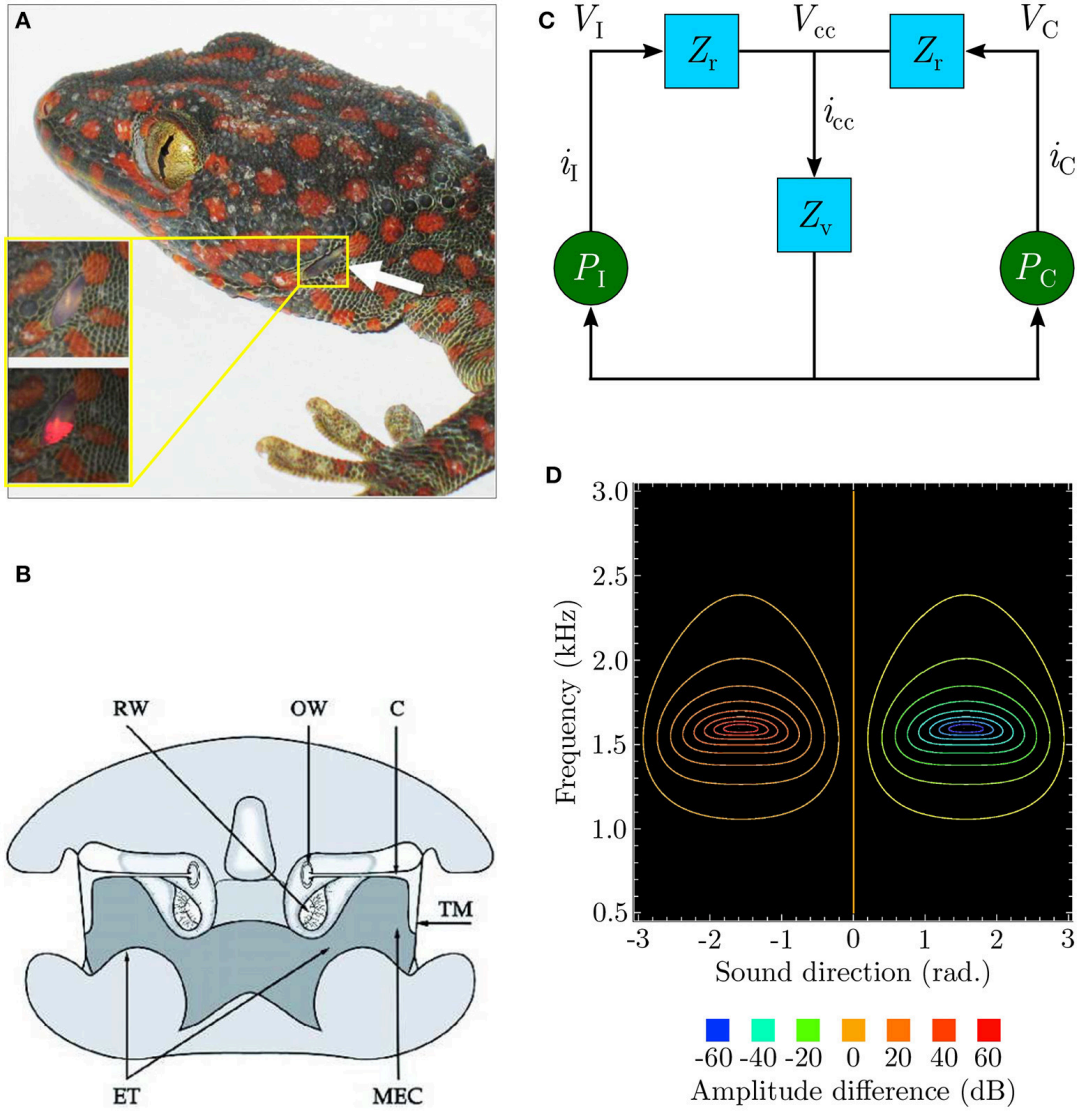
**(A)** An eardrum visible on the side of the gecko head (redrawn from Christensen-Dalsgaard et al., 2011). **(B)** Early cross-sectional diagram of the lizard (*Sceloporus*) auditory system (taken from Christensen-Dalsgaard and Manley, 2005). **(C)** Ideal lumped-parameter circuit model (based on Fletcher and Thwaites, 1979; Fletcher, 1992 and redrawn from Zhang, 2009). Voltages *V*_I_ and *V*_C_ respectively represent sound pressures *P*_I_ and *P*_C_ at the ipsilateral and contralateral eardrums. Currents *i*_I_ and *i*_C_, respectively represent the vibrations of the ipsilateral and contralateral eardrums due to the sound pressures acting upon them. Impedances *Z*_r_ model the combined acoustic filtering due to the mass of the eardrums and stiffness of the Eustachian tube through the central cavity connecting the tympani to each other. Impedance *Z*_v_ models the acoustic filtering effects of the central cavity itself. Voltage *V*_cc_ represents the resultant sound pressure in the central cavity due to the interaction of the internal sound pressures experienced from either side. This causes current *i*_cc_ to flow, representing the movement of sound waves inside the central cavity as the pressure inside it varies. **(D)** Contour plot (redrawn from Zhang, 2009) modelling binaural subtraction of the ipsilateral and contralateral responses as defined by Equation (2).

The remarkable sensitivity of the peripheral auditory system (Christensen-Dalsgaard and Manley, 2005; Christensen-Dalsgaard et al., 2011) of lizards such as the bronze grass skink or *Mabuya macularia*, and the tokay gecko or *Gekko gecko* as depicted in Figure 1A is quite well understood. This “directionality” enables the animal to extract the relative position of a relevant sound signal. The lizard ear achieves a directionality higher than that of any known vertebrate (Christensen-Dalsgaard and Manley, 2005). This is due to an internal acoustical connection formed by efficient sound transmission through internal pathways in the head as depicted in Figure 1B, between the animal's two eardrums.

In spite of the peripheral auditory system's relatively small dimensions (the eardrums for most lizard species are separated by 10–20 mm), the range of sound wavelengths over which it exhibits strong directionality (Christensen-Dalsgaard et al., 2011) is relatively wide (340–85 mm, corresponding to 1–4 kHz). Within this range of frequencies the sound pressure difference between the eardrums is negligible due to acoustic diffraction around the animal's head, thus generating almost negligible (1–2 dB) ILD cues. The system thus relies on μs-scale interaural phase differences between incoming sound waves at the two ears due to the physical separation. These phase differences, corresponding to ITDs, are used extract information about sound direction relative to the animal. The system essentially converts these relatively tiny phase differences into relatively larger (up to 40 dB) interaural vibrational amplitude differences (Christensen-Dalsgaard and Manley, 2005). These amplitude differences encode sound direction information. Each eardrum's vibrations are the result of the superposition of two acoustic components generated due to sound interference in the internal pathways–an external sound pressure acting on the eardrum's periphery and an equivalent internal sound pressure acting on its interior. This leads to the ipsilateral (toward the sound signal) amplification of eardrum vibrations and contralateral (away from the sound signal) cancellation of eardrum vibrations. In other words, the ear closer to the relevant sound signal vibrates more strongly as compared to the ear further away from it. The relative phase difference between the incoming sound waves at the two eardrums determines the relative strengths of their vibrations.

An equivalent electrical circuit model of the peripheral auditory system as depicted in Figure 1C (Fletcher and Thwaites, 1979; Fletcher, 1992) allows the directionality to be visualised as shown in Figure 1D as a difference signal computed by subtracting the vibrational amplitudes of the eardrums. Labelling the vibrational amplitudes of the ipsilateral and contralateral eardrums respectively as *i*_I_ and *i*_C_, the difference signal can be formulated as

(1) $$\begin{array}{l} |\frac{i_{\text{I}}}{i_{\text{C}}}|=|\frac{G_{\text{I}}\cdot V_{\text{I}}+G_{\text{C}}\cdot V_{\text{C}}}{G_{\text{C}}\cdot V_{\text{I}}+G_{\text{I}}\cdot V_{\text{C}}}|, \end{array}$$

where frequency-dependent gains *G*_I_ and *G*_C_ respectively model the effect of sound pressure on the motion of the ipsilateral and contralateral eardrum. These gains are essentially analogue filters in signal processing terminology with their coefficients determined experimentally from eardrum vibration measurements for individual lizards via laser vibrometry (Christensen-Dalsgaard and Manley, 2005). Expressing *i*_I_ and *i*_C_ in decibels,

(2) $$\begin{array}{l} i_{\text{ratio}}=20\left(\log|i_{\text{I}}|-\log|i_{\text{C}}|\right)\text{dB}. \end{array}$$

The model responds well for sound frequencies within the range 1–2.2 kHz, with a peak response at approximately 1.6 kHz. *i*_ratio_ is positive for |*i*_I_| > |*i*_C_| and negative for |*i*_C_| > |*i*_I_|. The model's symmetry implies that the model's response |*i*_ratio_| is identical on either side of the centre point θ = 0° as well as locally symmetrical within the sound direction range [−90°, +90°] (considered henceforth as the range of interest of sound direction). The difference signal expressed as Equation (2) provides information about sound direction in that its sign indicates whether the sound is coming from the ipsilateral side (positive sign) or from the contralateral side (negative sign), while its magnitude corresponds to the relative angular displacement of the sound signal with respect to the median.

### 2.2. Input correlation (ICO) learning

**Figure 2:**
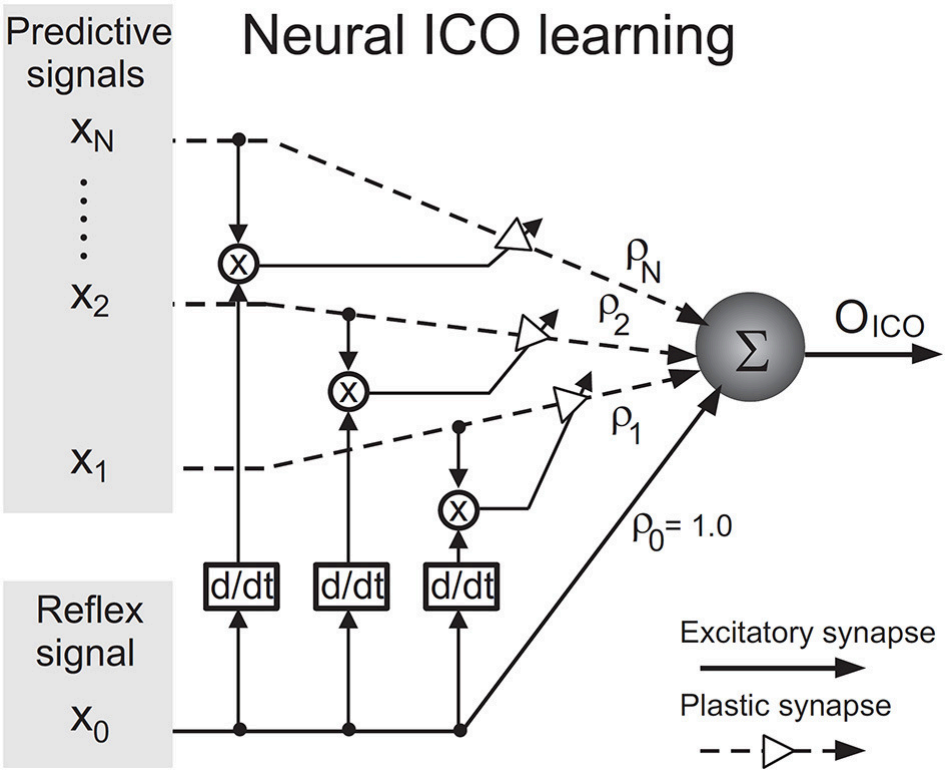
**Neural circuit for input correlation learning (taken from Manoonpong et al., 2013)**.

Since the proposed neural mechanism is derived from the ICO learning algorithm (Porr and Wörgötter, 2006), this section gives a brief introduction to the algorithm. The algorithm, depicted as a neural mechanism in Figure 2, is online unsupervised learning. Its synaptic weight update is driven by cross-correlation of two types of input signals–one or multiple “predictive” signal(s) which are stimuli occurring earlier in time and a “reflex” signal which is a stimulus occurring later in time, that arrives after a finite delay and drives an unwanted response or reflex. The learning goal of ICO learning is to predict the occurrence of the reflex signal by utilising the predictive signal. This allows an agent to react earlier, before the reflex signal occurs. The agent essentially learns to execute an anticipatory action to avoid the reflex.

The output *O*_ICO_ of the ICO learning mechanism is a linear combination of the reflex input *x*_0_ and the *N* predictive input(s) *x*_k_ where k = 1, …, *N* and *N* ∈ ℕ. *O*_ICO_ is formulated as

(3) $$\begin{array}{l} O_{\text{ICO}}=\rho_{0}x_{0}\left(t\right)+\overset{N}{\underset{\text{k}=1}{\sum }}\rho_{\text{k}}\left(t\right)x_{\text{k}}\left(t\right). \end{array}$$

The synaptic weight ρ_0_ of the reflex input is assigned a constant positive value such as 1.0, representing a reflex signal whose strength does not change over time. During learning, the synaptic weight(s) ρ_k_ of the predictive signal(s) *x*_k_(*t*) are updated through differential Hebbian learning (Kosko, 1986; Klopf, 1988) using the cross-correlation between the predictive and reflex inputs. The synaptic weight update rule is given by

(4) $$\begin{array}{l} \frac{d\rho_{\text{k}}\left(t\right)}{dt}=\mu x_{\text{k}}\left(t\right)\frac{dx_{0}\left(t\right)}{dt},\text{k}=1,\ldots ,N. \end{array}$$

The learning rate μ, usually set to a value less than 1.0, determines how fast the neural mechanism can learn to avoid the reflex signal from occurring. The synaptic weights ρ_k_ tend to stabilise when the reflex signal is nullified, which implies that the reflex signal has been successfully avoided. ICO learning is characterised by its fast learning speed and stability of synaptic weight updates and has been successfully applied to real robots to generate adaptive behaviour (Manoonpong et al., 2007; Porr and Wörgötter, 2007; Manoonpong and Wörgötter, 2009).

## 3. Materials and methods

We define the task of acoustic tracking as follows–a robotic agent must learn to track a moving acoustic signal. The robot learns the target's angular velocity by matching it with its (the robot's) own angular turning velocity. The correct angular turning velocity should allow the agent to rotate along a fixed axis sufficiently quickly so as to align itself toward the instantaneous position of the acoustic signal. The signal is moved in the horizontal plane along a pre-defined semi-circular arc-shaped trajectory with an unknown velocity in an unknown but fixed direction. To solve this task we employ an adaptive neural architecture (Shaikh and Manoonpong, 2016) that combines the auditory preprocessing of the lizard peripheral auditory model with a neural ICO-based learning mechanism.

### 3.1. The neural architecture

**Figure 3:**
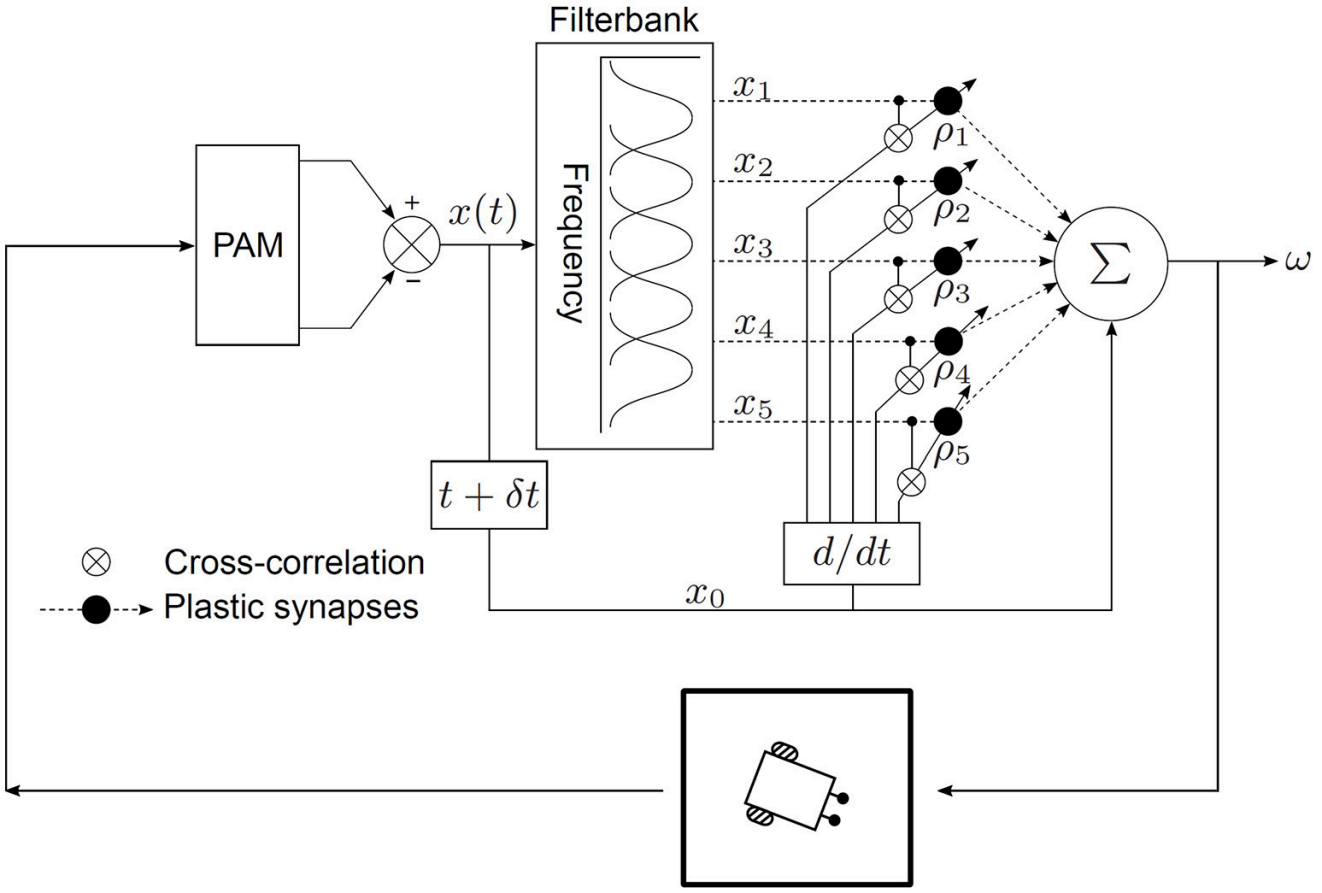
**Neural mechanism for acoustic tracking as a closed-loop system**.

The neural mechanism is embedded within the task environment as a closed-loop circuit as depicted in Figure 3. The goal of the learning algorithm is to learn the temporal relationship between the perceived position of the target sound signal *before* turning and *after* turning. The synaptic weights of the neural mechanism encode this temporal relationship and they can then be used to calculate the correct angular turning velocity. A given set of learned synaptic weights can however only represent a given angular velocity. This is because the temporal relationship between the perceived position of the target sound signal *before* turning and *after* turning depends on the angular turning velocity. Therefore, the synaptic weights must be re-learned to obtain a new angular turning velocity.

The output of the neural mechanism is the angular velocity ω, defined as the angular deviation per time step, required to turn the robot quickly enough to orient toward the target sound signal in one time step. The rotational movements of the robotic agent translate ω into corresponding ITD cues. The peripheral auditory model (PAM), based on these cues, computes a difference signal *x*(*t*) which encodes information regarding sound direction. Practically, *x*(*t*) is the difference between the modelled vibrational amplitudes of the left and right eardrums in response to sound input, i.e., it is essentially *i*_ratio_ as defined by Equation (2). A filter bank decomposes *x*(*t*) into sound frequency-dependent components *x*_k_(*t*), where k = 1, …, *N*, to extract frequency information. Each of these components encodes the extracted sound direction information within a specific frequency band. Practically, these components are the difference between the modelled vibrational amplitudes of the left and right eardrums in response to sound input. This step is necessary since the peripheral auditory model provides ambiguous information regarding the sound direction in the absence of sound frequency information. The ambiguity is a result of the difference signal *x*(*t*) having identical values for multiple positions of the sound signal if the sound frequency is unknown (see Figure 1D). The filter bank comprises five bandpass filters. The centre frequencies of these filters lie at 1.2, 1.4, 1.6, 1.8, and 2.0 kHz within the relevant response range. Each filter has a 3 dB cut-off frequency of 200 Hz. This results in *N* = 5 filtered difference signals at the output of the filter bank. The magnitude responses of the individual filters in the filter bank represent the receptive fields of individual auditory neurons. These spectro-temporal receptive fields (Aertsen et al., 1980) are essentially the range of sound frequencies over which the neurons are optimally stimulated. The filtered difference signals *x*_k_(*t*) are then used as inputs that are correlated with the derivative of the unfiltered difference signal *x*_0_(*t*). The input signals *x*_k_(*t*) represent the earlier-occurring predictive stimuli used to estimate the instantaneous sound direction before turning, while the unfiltered difference signal *x*_0_(*t*) represents the later-occurring “reflex” stimuli or the retrospective signal generated after turning.

In traditional ICO learning the synaptic weights are stabilised once the reflex signal is nullified, thereby creating a behavioural response that prevents future occurrences of the reflex signal. In our case, as soon as the target sound signal moves to a new position along its trajectory, a new and finite retrospective signal *x*_0_ corresponding to the new position is generated. This signal is then nullified after turning if the correct synaptic weights have been learned, and then the target sound signal moves to a new position along its trajectory. Our approach can therefore be considered as one successful step of ICO learning being successively repeated for each new position of the target sound signal as it moves along its trajectory. This implies that the synaptic weights can grow uncontrollably if the learning is allowed to continue indefinitely. A stopping criterion for the learning was therefore introduced to avoid this condition–the learning stops when the tracking error θ_*e*_ becomes less than 0.5°. θ_*e*_ is defined as the difference between the orientation of the robot and the angular position of the sound signal in *one* time step. In other words, the learning stops when the robot is able to orient itself toward a position that is within 0.5° from the position of the sound signal within *one* time step.

### 3.2. The experimental setup

**Figure 4:**
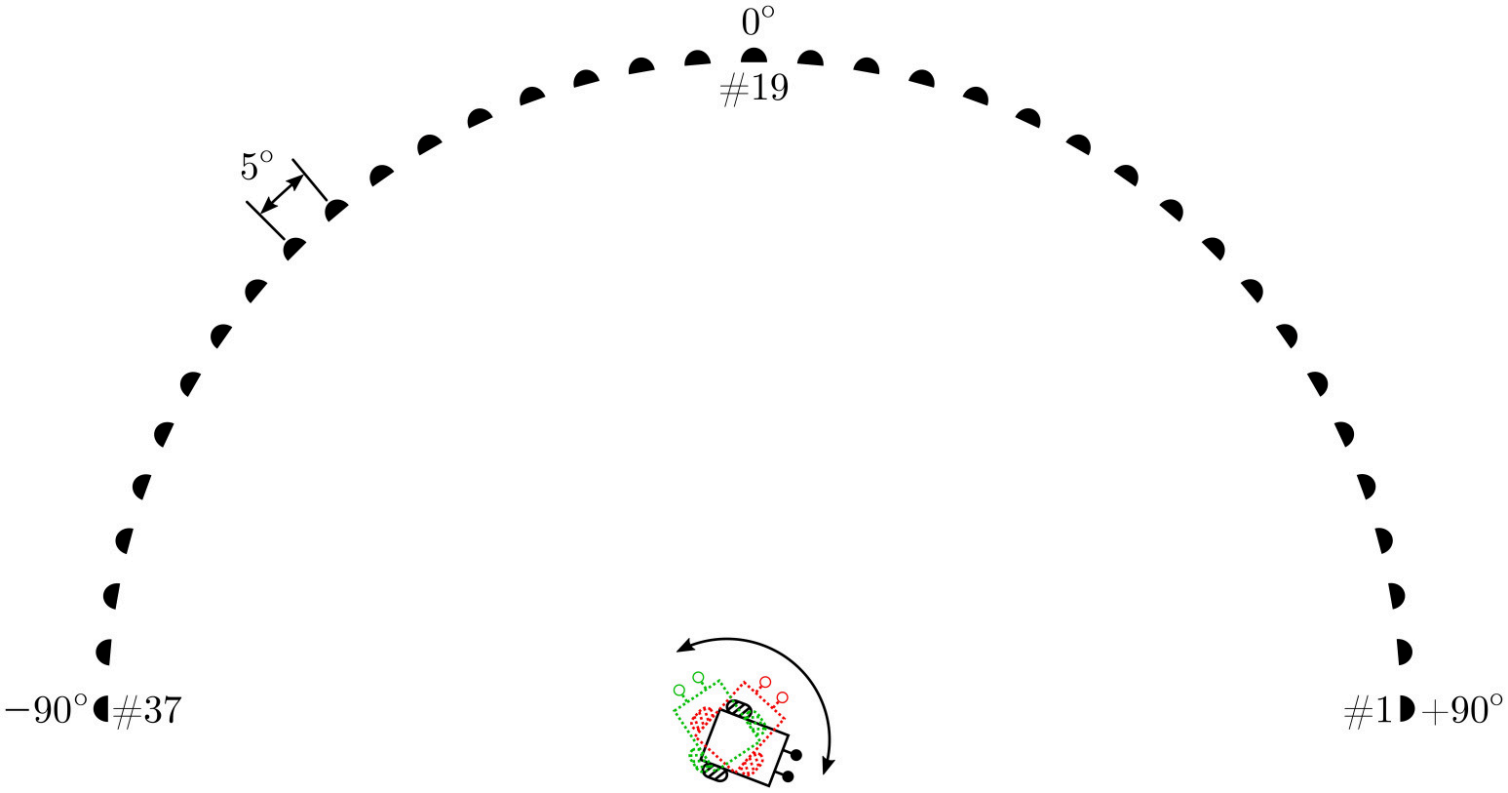
**The simulation setup**.

The experimental setup in simulation comprises a virtual loudspeaker array as depicted in Figure 4 which generates relevant pure tone sounds at a 2.2 kHz frequency. This frequency is chosen because sufficient directional information from the peripheral auditory model is available at this frequency. The array consists of 37 loudspeakers numbered #1–#37 from right to left, arranged in a semi-circle in the azimuth plane. The angular displacement between consecutive loudspeakers is 5°. The loudspeakers are turned on sequentially, starting from the loudspeaker at one of the ends of the array, to simulate the motion of a continuously moving sound signal (albeit in discrete steps). To maintain the continuity of the sound the next loudspeaker plays immediately after the previous loudspeaker has stopped. A given tone can therefore be moved with a given angular velocity across the array along a semi-circular trajectory from either the left or the right side. The angular velocity of the sound signal is defined as the angular displacement in radians per time step. A given loudspeaker, when turned on, plays a tone for 10 time steps before turning off and at the same instant the next consecutive loudspeaker turns on. This process is repeated until the sound reaches the last loudspeaker in the array. The movement of sound from loudspeaker #1 to loudspeaker #37 is defined as one complete learning iteration. Since one iteration may be insufficient to learn the correct angular velocity of the target sound signal, the learning is repeated over multiple iterations until the stopping criterion is met. After the completion of one learning iteration, the sound signal starts again from loudspeaker #1 in the next learning iteration. The direction of movement of sound is chosen to be from the right side (+90°) to the left side (−90°) of the array.

The robot that should track the moving target sound signal is positioned at the mid-point of the diameter of the semi-circle and is only allowed to rotate in the azimuth plane along a fixed axis. The robot must turn with a sufficiently large angular turning velocity to orient toward the instantaneous position of sound signal before the sound signal moves to the next position along its trajectory. The angular velocity of the robot is defined as the angular rotation in radians per time step. The goal of the learning algorithm is to learn the correct angular velocity that allows the robot to turn and orient toward the current loudspeaker in *one* time step, starting from the time step at which that loudspeaker started playing the tone.

The learning at every time step occurs as follows. The robotic agent is initially oriented toward a random direction toward the right side of the array. Loudspeaker #1 emits a tone and the robot uses the sound direction information extracted by the peripheral auditory model to turn toward the currently playing loudspeaker with an angular velocity ω (computed using the initial values of the synaptic weights) given by

(5) $$\begin{array}{l} \omega =\rho_{0}x_{0}+\overset{N}{\underset{\text{k}=1}{\sum }}\rho_{\text{k}}x_{\text{k}},\text{where }N=5. \end{array}$$

After the turn is complete, the robot once again extracts sound direction information via the peripheral auditory model and computes the retrospective signal *x*_0_(*t* + δ*t*). The strength of *x*_0_(*t* + δ*t*) depends on the relative position of the sound signal with respect to the orientation of the robotic agent after it has performed a motor action in the task environment. Therefore, this retrospective signal acts as the feedback information that is used to update the synaptic weights.

The synaptic weights ρ_k_ are then updated according to the learning rule

(6) $$\begin{array}{l} \frac{d\rho_{\text{k}}\left(t\right)}{dt}=\mu x_{\text{k}}\left(t\right)\frac{dx_{0}\left(t\right)}{dt},\text{where k}=1,\ldots ,N. \end{array}$$

After 10 time steps loudspeaker #1 is deselected and the next loudspeaker in the array (loudspeaker #2) is selected. This learning procedure is repeated for all loudspeakers in succession.

We use three different angular velocities for the sound signals–0.5°/time step, 1.0°/time step and 1.5°/time step. These values were chosen primarily because the loudspeaker array in the experimental setup in practice is restricted to sound signal displacements that are multiples of 5°. The neural parameters for all trials are set to the following values–the learning rate μ = 0.0001 and synaptic weight for the retrospective signal *x*_0_, ρ_0_ = 0.00001. All plastic synaptic weights ρ_k_ are initially set to zero and updated according to Equation (6).

**Figure 5:**
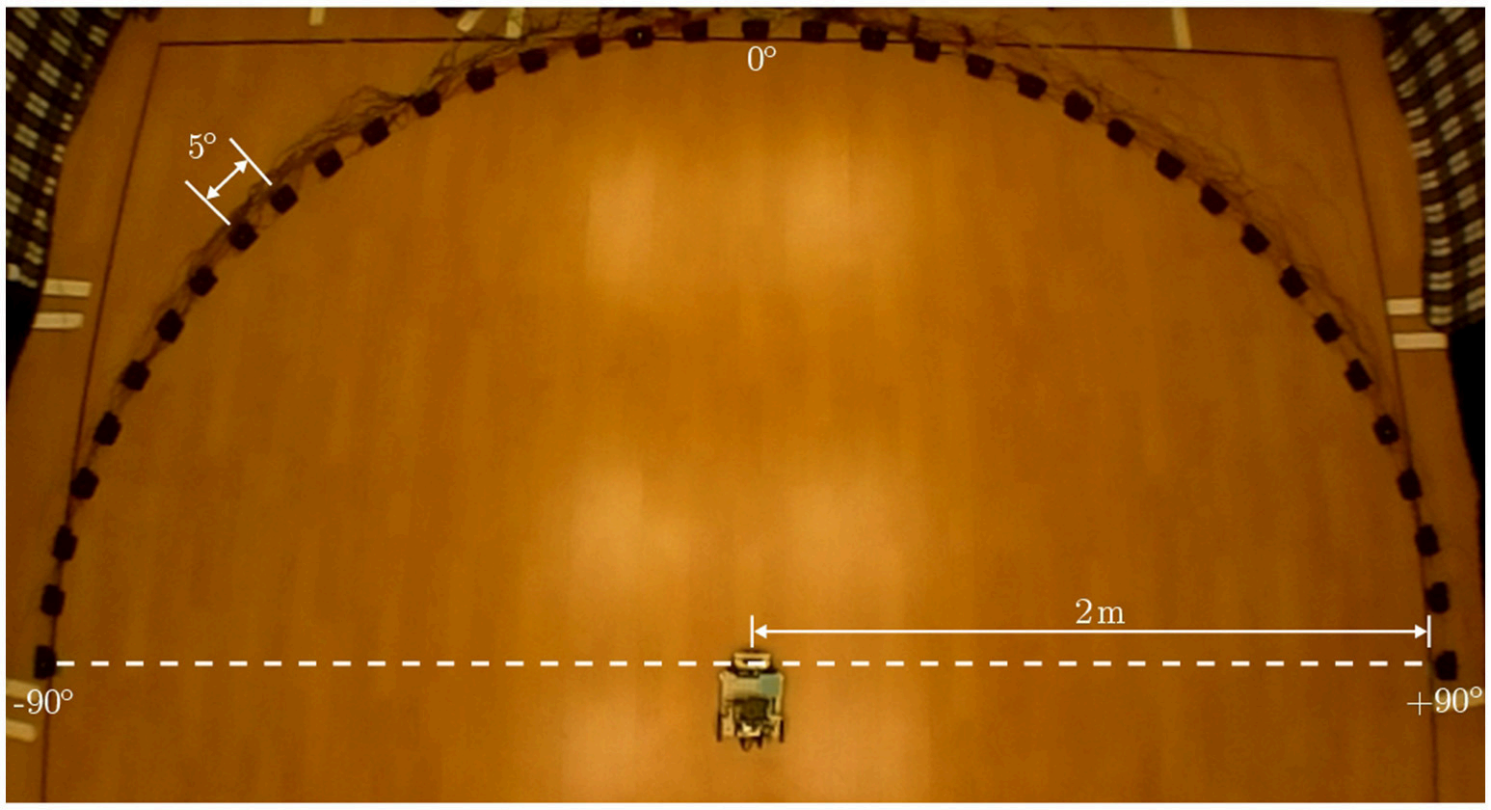
**The experimental arena**.

We first implement a new instance of the neural learning mechanism in simulation. The mechanism allows a robotic agent to learn the synaptic weights required to track a continuous unoccluded sound signal in simulation. The initial orientation of the robotic agent is randomly chosen to be 116° to emphasise that the learning is independent of any specific initial orientation. The continuous unoccluded sound can be viewed as a sound with 100% sound emission duty cycle, i.e., there are no breaks in the sound emission. We evaluate the acoustic tracking performance in simulation for a set of three different target angular velocities–0.5°/time step, 1.0°/ time step and 1.5°/time step. We then verify the simulation results in practice for a target angular velocity of 1.5°/time step by recreating the experimental setup in the form of robotic trials. We employ a wheeled mobile robot, as described in Section 3.3, to track a continuous unoccluded pure tone sound signal that is moved along a semi-circular virtual loudspeaker array as depicted in Figure 5. The array has an identical configuration as the one used in the simulation setup and is located in a sound-dampening chamber to minimise acoustic reflections. The synaptic weights used on the robot are those learned offline in simulation.

We then use another identical instance of the neural mechanism in the same simulation setup as before to learn to track a virtual pure tone sound signal that is periodically occluded, i.e., it is structured as a constant sound for a constant interval followed by complete silence for a constant interval. This sound emission duty cycle is set to 60%. The target sound signal again moves with a constant but unknown angular velocity along a semi-circular trajectory as described earlier. The initial orientation of the robotic agent in simulation is randomly chosen to be 97° to emphasise that the learning is independent of any specific initial orientation. In this manner, the acoustic tracking performance is evaluated in simulation for a set of three different target angular velocities–0.5°/time step, 1.0°/time step and 1.5°/time step. The simulation results are validated for a target angular velocity of 1.5°/time step in practice via robotic trials with the mobile robot as described earlier. The synaptic weights used on the robot are again those learned offline in simulation.

Finally, we use a third instance of the neural mechanism in simulation to learn to track a virtual pure tone sound signal that is occluded as described earlier but with a randomly varying duty cycle of sound emission. During learning, for every loudspeaker the sound emission duty cycle is chosen from a uniform random distribution between 10 and 90%. As before, the target sound signal moves with a constant but unknown angular velocity along a semi-circular trajectory as described earlier. The initial orientation of the robotic agent in simulation is randomly chosen to be 97°, once again to emphasise that the learning is independent of any specific initial orientation. We evaluate the acoustic tracking performance in simulation for a set of three different target angular velocities–0.5°/time step, 1.0°/time step and 1.5°/time step. We once again validate the simulation results in practice for a target angular velocity of 1.5°/time step on the mobile robot as described earlier. The sound emission duty cycles for each loudspeaker in the robotic trial are again randomly chosen from a uniform random distribution between 10 and 90%. This implies that the sequence of duty cycles is not identical to that used in the simulated trials. As earlier, the synaptic weights used on the robot are those learned offline in simulation.

### 3.3. The robot model

**Figure 6:**
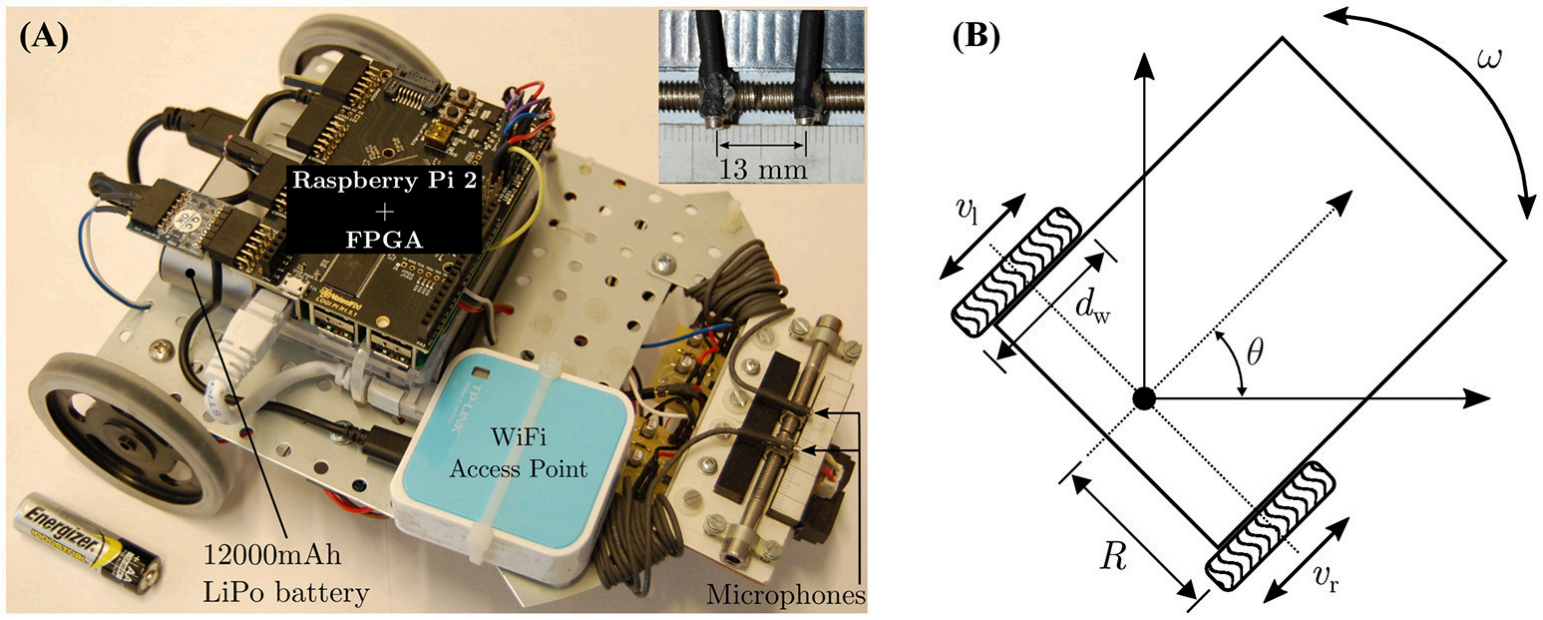
**(A)** The mobile robot and **(B)** its kinematics.

Figures 6A,B respectively depict the mobile robot used in the robotic trials and its kinematics. The basic platform is assembled with components from the Robotics Starter Kit from Digilent Inc.–the chassis, the DC motors (6 V), the corresponding H-bridge motor drivers, the rear wheels and a front omnidirectional ball caster wheel. The peripheral auditory model and the neural mechanism is implemented on a Raspberry Pi 2 (Model B+ from the Raspberry Pi Foundation) controller, which is paired with a FPGA board (model LOGI Pi from ValentFX). A dual channel analogue-to-digital (ADC) driver is implemented on the FPGA IC (Integrated Circuit) using the VHDL (VHSIC Hardware Description Language) programming language (VHSIC stands for Very-High-Speed Integrated Circuits). The VHDL design for the ADC driver is synthesised or compiled via a proprietary software tool (Xilinx Integrated Synthesis Environment or ISE from Xilinx Inc.) into a hardware-level binary “bitstream” containing all the necessary information to properly configure and program the logic into the FPGA chip. The driver reads in raw audio data from a dual channel 12-bit simultaneous ADC that digitises the signals from two omnidirectional microphones (model FG-23329-P07 from Knowles Electronics LLC) mounted 13 mm apart at the front of the robot (see inset in Figure 6). Since the peripheral auditory model's parameters have been derived from laser vibrometry measurements from a lizard with 13 mm separation between its eardrums, the microphone separation must match that value. Any other separation would create a mismatch between the ITD cues to which the peripheral auditory model is tuned and the actual ITD cues. A WiFi access point (model TL-WR802N from TP-LINK Technologies Co. Ltd.) allows wireless access to the robot controller for programming purposes. A 12,000 mAh lithium polymer power bank (model Xtorm AL450 from A-solar bv) serves as the power source for the robot.

The robot's kinematics are used to convert the learned angular rotation in degrees per second into the rotational speed in revolutions per minute (rpm) for the robot's wheels. One time step in simulation corresponds to 0.2 s, such that a learned angular turning velocity of θ degrees per time step implies that the robot should turn by θ degrees in a time period *t*_θ_ = 0.2 s. This value is chosen because in the experimental setup the software controlling the loudspeaker array can only switch between consecutive loudspeakers at least every 2 s. Since the robot can only rotate along a fixed axis, the wheels travel along a semi-circular arc of length $\overset{⌢}{\text{L}}$ when the robot performs a turn. Therefore, an angular displacement of θ degrees corresponds to the arc length $\overset{⌢}{\text{L}}$ in millimetres as given by

(7) $$\begin{array}{l} \overset{⌢}{\text{L}}=2\pi R\frac{\theta }{360^{{}^{\circ}}}, \end{array}$$

where *R* is the radius in millimetres of the arc along which the wheels travel, and is essentially the distance between the centre of rotation of the robot and the centre of either wheel. Assuming *v*_l_ and *v*_r_ as the rotational velocities of the left and right wheels respectively, to rotate through an arc length $\overset{⌢}{\text{L}}$ the two wheels must turn with identical angular velocities |*v*_l_| = |*v*_r_| = *v* but in opposite directions (to perform a leftward rotation, *v*_l_ is considered as having a negative value and *v*_r_ is considered as having a positive value). The angular velocity $\omega =\frac{\overset{⌢}{\text{L}}}{t_{\theta }}$ mm/s. The wheel rotational velocity *v* in rpm is given by

(8) $$\begin{array}{l} \begin{array}{c} v=\frac{\overset{⌢}{\text{L}}}{t_{\theta }}\cdot \frac{1}{\pi d_{\text{w}}}\cdot 60\text{ s}\\ =2\pi R\frac{\theta }{360^{{}^{\circ}}}\cdot \frac{1}{t_{\theta }}\cdot \frac{1}{\pi d_{\text{w}}}\cdot 60\text{ s}, \end{array} \end{array}$$

where *d*_w_ is the diameter of the wheel. For the robot, *R* is measured to be 80 mm and *d*_w_ is measured to be 70 mm. Substituting for *R*, *d*_w_ and *t*_θ_ into Equation (8), the mathematical conversion between the robot's angular velocity in degrees per second into the corresponding wheel velocity *v* in rpm can be formulated as

(9) $$\begin{array}{l} \begin{array}{c} v=2\pi \cdot 80\text{ mm}\cdot \frac{\theta }{360^{{}^{\circ}}}\cdot \frac{1}{0.2\text{ s}}\cdot \frac{1}{\pi 70}\cdot 60\text{ s}\approx 3.81\cdot \theta . \end{array} \end{array}$$

Using Equation (9), the wheel velocities required by the robot corresponding to the three angular velocities 0.5°/time step, 1.0°/time step and 1.5°/time step are calculated to be approximately 19 rpm, 38 rpm and 57 rpm respectively. These rpm values represent the no-load wheel velocities, i.e., when the DC motor shafts experience zero load. In practice these “ideal” rpm values will be adversely affected by the weight of the robot, the friction between the wheels and the ground and the instantaneous battery capacity. To approach real-life motion constraints during tracking, the effects of these physical quantities are deliberately not modelled. The speed commands for the wheels are therefore manually matched to the corresponding wheel velocities under load. This is done by making the robot perform an on-the-spot turn on the ground in the experimental arena, and determining via trial and error the speed command (which is the duty cycle for the signals controlling the motor drivers) for which the wheels complete the necessary revolutions in 1 min. This ensures that the effects of the aforementioned quantities are taken into account while the robot is tracking the sound signal during the robotic trials. Furthermore, there may be a mismatch between the characteristics of the individual DC motors of the robot. This may result in a mismatch between the angular velocities of the motor shafts even though both motors receive identical speed commands. To compensate for any potential mismatch, the robot is once again made to perform on-the-spot turns on the arena floor and the speed commands were fine-tuned via trial and error to generate turns of 0.5°, 1.0°, and 1.5° in 0.2 s.

**Figure 7:**
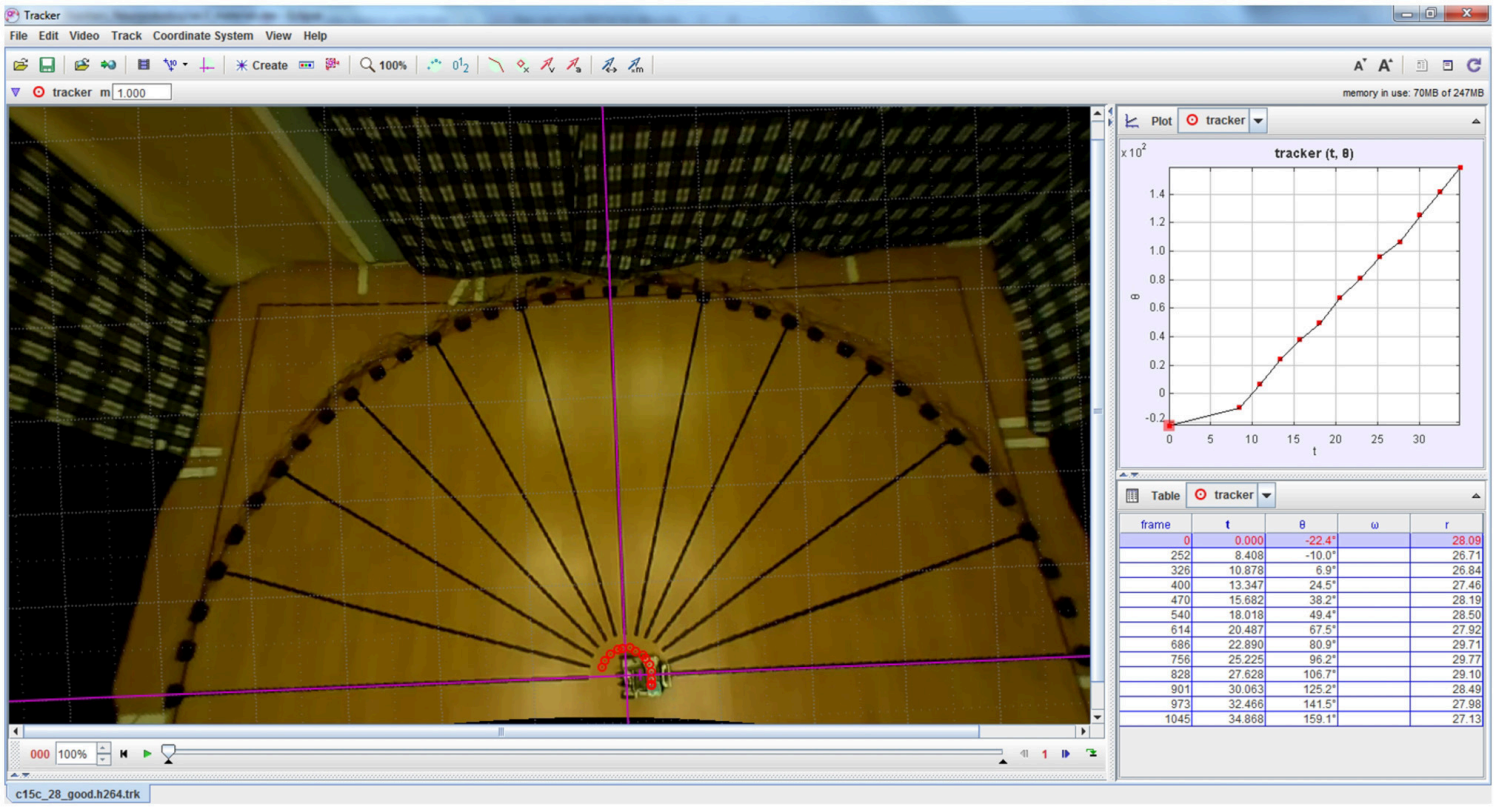
**Example screenshot of Tracker software used for extracting the robot's turning angles from overhead video footage of the robotic trials**. The red circles indicate the location of the LED on the robot that is used for computing its angular rotation.

Video footage of the robotic trials was recorded from an overhead camera (Raspberry Pi camera module from the Raspberry Pi Foundation). The footage was analysed with a video analysis software tool (Tracker version 4.95 from Open Source Physics (Open Source Physics, 2016) to determine the amount by which the robot turned for each loudspeaker. The robot's rotation angles were extracted by manually tracking a green LED (Light Emitting Diode) on the robot. The tracking was done for relevant video frames in which the robot was completely still after completing a turn, to determine its deviation from the reference. Figure 7 depicts a screenshot of Tracker software.

## 4. Results and discussion

### 4.1. Simulation trials

**Figure 8:**
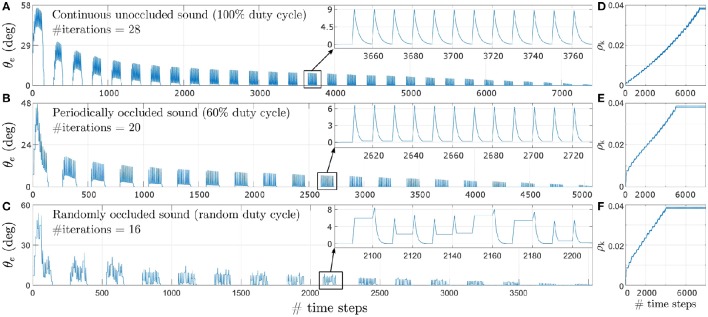
**Tracking error θ_*e*_ for a target angular velocity of 1.5°/time step for varying duty cycles of sound emission. (A)** Continuous unoccluded sound. **(B)** Periodically occluded sound with 60% duty cycle. **(C)** Randomly occluded sound with random duty cycle. The insets show θ_*e*_ for a single iteration as an example. **(D)** Synaptic weights for continuous unoccluded sound. **(E)** Synaptic weights for periodically occluded sound with 60% duty cycle. **(F)** Synaptic weights for randomly occluded sound with random duty cycle.

Figure 8 depicts the evolution of the tracking error θ_*e*_ during learning for a target angular velocity of 1.5°/time step as an example. Corresponding data for target angular velocities of 1.0°/time step and 0.5°/time step is illustrated respectively in Figures 8-1, 8-2 (see files “image1.pdf” and “image2.pdf” respectively in the Supplementary Materials). The insets show θ_*e*_ for a single iteration as an example. θ_*e*_ reduces exponentially over time for all three types of acoustic stimuli–continuous unoccluded sound (see Figure 8A), periodically occluded sound with a 60% sound emission duty cycle (see Figure 8B) and randomly occluded sound with a random sound emission duty cycle (see Figure 8C).

The spikes in θ_*e*_ as visible in the insets are a result of a mismatch between the last angular position toward which the robotic agent was pointing and the new angular position of the target sound signal as it moves along its trajectory. This mismatch generates finite ITD cues from which the robotic agent extracts sound direction information using the peripheral auditory model. The robotic agent then turns toward the sound signal with the last learned angular turning velocity, thereby reducing the maximum tracking error. This process repeats for each time step, exponentially reducing the overall tracking error, until the stop criterion is met.

The number of iterations required for the synaptic weights to converge toward their final values is relatively lower for sparse or occluded sound signals as compared to unoccluded sound signals. For an occluded signal, the number of time steps for which the sound is emitted per loudspeaker decreases. Consequently, the number of weight updates per loudspeaker also decreases. For example, for a 60% duty cycle, the weights are updated is six of the ten time steps per loudspeaker. When the loudspeaker stops playing there is no sound input and the peripheral auditory model's outputs are balanced, resulting in the difference signal *x*(*t*) being nullified. This implies that both the retrospective signal *x*_0_(*t*) and the predictive signal *x*_k_(*t*) become zero. The weight increment given by the update rule in Equation (6) is therefore also zero when there is no sound present. From the perspective of the robotic agent's behaviour, this implies that the robotic agent does not move in the absence of sound. This is because there is no directional information available and the robotic agent “assumes” that it is already oriented toward the target sound position. Thus for occluded sound signals the robotic agent takes relatively fewer turns for each loudspeaker as compared to the number of turns taken for each loudspeaker for the unoccluded sound signal. This means that when the sound moves to a new position along its trajectory, the tracking error is relatively larger for occluded sound signals as compared to the unoccluded sound signal. This implies a relatively greater mismatch between the actual angular position of the loudspeaker and the orientation of the robotic agent and therefore relatively larger values for both the predictive and retrospective signals. Consequently, the synaptic weight update is also relatively larger for occluded sound signals as compared to unoccluded sound signal from the very first iteration as evident from Figures 8D,E. These large changes at the very beginning of the learning bring the synaptic weights relatively closer to their optimal values earlier in the learning process, and thus fewer subsequent iterations are required to bring the weights to their optimal values. Therefore, for a given target angular velocity the total number of iterations required for the synaptic weights to converge decreases for occluded sound signals as compared to the unoccluded sound signal.

The change in tracking error θ_*e*_ for a pure tone sound signal that is randomly occluded with a sound emission duty cycle between 10 and 90% for each loudspeaker is depicted in Figure 8C for the target angular velocity of 1.5°/time step. The insets show θ_*e*_ for a single iteration as an example. The uneven spikes visible in the insets indicate that the tracking error θ_*e*_ is different for different angular positions of the target sound signal. For each new angular position of the target sound signal, the learning algorithm increments the synaptic weights corresponding to the randomly selected sound emission duty cycle currently in effect for that angular position. Therefore, the synaptic weight increments are also random as evident in Figure 8F. As discussed earlier, a relatively smaller sound emission duty cycle results in relatively fewer weight updates. This implies that when the current duty cycle is relatively small, the robotic agent makes relatively fewer turns and thus the tracking error may only decrease to a finite non-zero value. When the target sound signal subsequently moves to the new consecutive angular position along its trajectory, the tracking error increases again due to mismatch between the last angular position estimated by the robotic agent and the new angular position of the target sound signal. The amount of mismatch depends on the last learned angular turning velocity of the robotic agent. This in turn depends on the sound emission duty cycle for the last target angular position and that for the current target angular position. If the new sound emission duty cycle is relatively larger than the last one then there are relatively more weight updates. The tracking error may either reduce to zero or to another finite but non-zero value for that particular target angular position.

**Figure 9:**
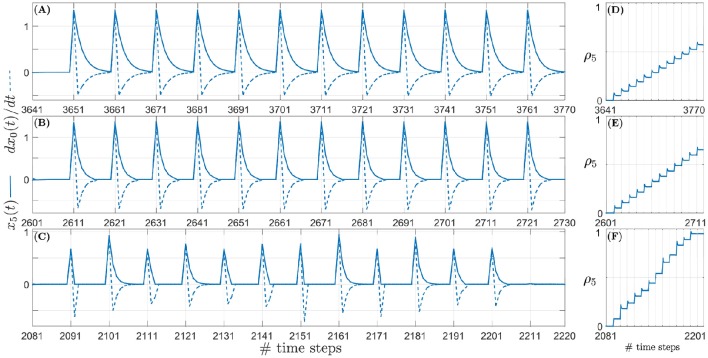
**Example snapshots of the synaptic weight updates (right column) corresponding to the correlation between the predictive signal *x*_5_(*t*) (solid line, left column) and the derivative of the retrospective signal $\frac{dx_{0}\left(t\right)}{dt}$ (dashed line, left column) for a sound signal moving with an angular velocity of 1.5°/time step. (A)** Continuous unoccluded sound. **(B)** Periodically occluded sound with 60% duty cycle. **(C)** Randomly occluded sound with random duty cycle. **(D)** Synaptic weights for continuous unoccluded sound. **(E)** Synaptic weights for periodically occluded sound with 60% duty cycle. **(F)** Synaptic weights for randomly occluded sound with random duty cycle.

As an example, the relationship between the predictive signal *x*_5_(*t*) and the derivative of the retrospective signal $\frac{dx_{0}\left(t\right)}{dt}$ and the corresponding weight updates can be seen in Figure 9 over one iteration of the learning. In the figure the sound signal is moving with an angular velocity of 1.5°/time step. Similar relationships corresponding to target angular velocities of 1.0°/time step and 0.5°/time step are illustrated respectively in Figures 9-1, 9-2 (see files “image3.pdf” and “image4.pdf” respectively in the Supplementary Materials). The respective weight updates (normalised for comparison) for all three types of acoustic stimuli–continuous unoccluded sound (see Figure 9D), periodically occluded sound with a 60 % sound emission duty cycle (see Figure 9E) and randomly occluded sound with a random sound emission duty cycle (see Figure 9F)–reflect the dependence of the size of the weight increments on the sound emission duty cycle as discussed earlier. The small initial spikes seen in the weight updates are a result of the $\frac{dx_{0}\left(t\right)}{dt}$ term in Equation (6) being initially positive and then becoming negative in the subsequent time step. The retrospective signal *x*_0_(*t*) is first positive due to the sound signal moving further away from the robotic agent. In the subsequent time step the robot reacts by turning toward the sound signal, thereby reducing *x*_0_(*t*). This results in the derivative $\frac{dx_{0}\left(t\right)}{dt}$ being negative. This leads to a negative weight increment which decreases the weight in the subsequent time steps after the spike. The term $\frac{dx_{0}\left(t\right)}{dt}$ becomes negative because the robot always turns toward the sound signal, which reduces *x*_0_(*t*).

**Figure 10:**
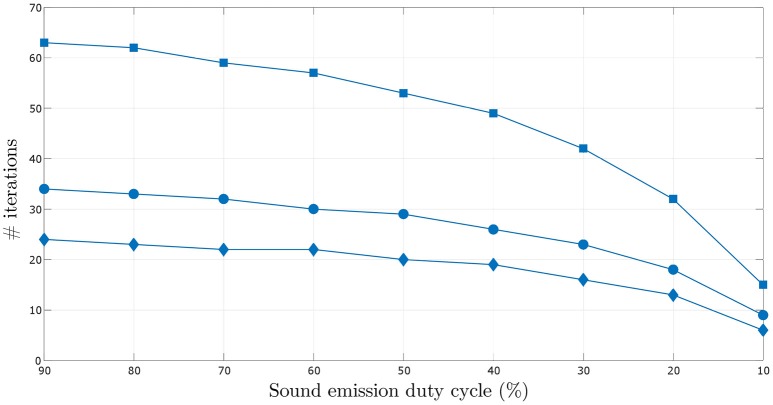
**Comparison of the number of iterations required for the synaptic weights to converge over varying sound emission duty cycles in the range 10–90%, for the three target angular velocities–0.5°/time step (square markers), 1.0°/time step (circular markers) and 1.5°/time step (diamond markers)**.

A more thorough investigation of the effect of decreasing sound emission duty cycle on the number of iterations required to learn the target angular velocity within the given error bounds is depicted in Figure 10. The number of iterations required for the synaptic weights to converge decreases with decreasing sound emission duty cycle, i.e., with increasing sparsity of sound stimulus as described earlier.

The number of iterations required for the synaptic weights to converge also decreases for increasing angular velocity of the sound signal. This can be seen in Figures 8-1, 8-2 (see files “image1.pdf” and “image2.pdf” respectively in the Supplementary Materials). For increasing target angular velocity, the mismatch between the angular position toward which the robot was oriented after its last turn and the current position of the sound signal is relatively greater. This results in relatively larger predictive signals *x*_k_(*t*), and therefore a relatively larger correlation term $x_{k}\left(t\right)\frac{dx_{0}\left(t\right)}{dt}$ per time step in Equation (6). This consequently leads to relatively faster weight updates, reducing the total number of time steps and thus iterations taken to learn the correct angular velocity.

### 4.2. Real robot implementation

Individual robotic trials are conducted for continuous unoccluded as well as occluded sound signals. In all trials, the sound signal is moved virtually in the experimental arena as depicted in Figure 5 with an angular velocity of 1.5°/0.2 s. We present video footage of the trials in which the robot's tracking behaviour after learning can be seen. Supplementary Videos #1, #2 and #3 (see files titled “video1.mp4”, “video2.mp4” and “video3.mp4” respectively in the Supplementary Materials) respectively show the tracking behaviour for the continuous unoccluded sound signal with a duty cycle of 100%, the periodically occluded signal with a duty cycle of 60% and the randomly occluded signal with a random duty cycle between 10 and 90% for each loudspeaker. As evident from the video footage, in all trials the robot is able to successfully perceive the acoustic motion of the sound signal and orient toward the currently playing loudspeaker as indicated by a green LED mounted on the top of the loudspeaker.

**Figure 11:**
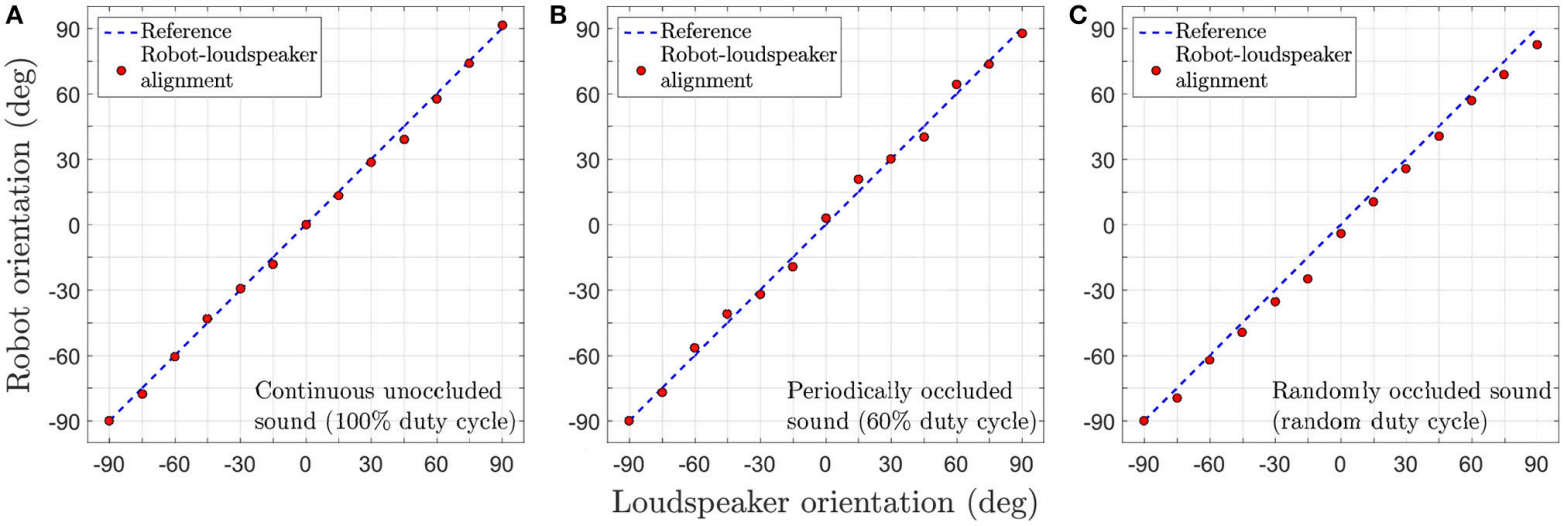
**Tracking performance during the robotic trials for a target angular velocity of 1.5°/time step. (A)** Continuous unoccluded sound. **(B)** Periodically occluded sound with 60% duty cycle. **(C)** Randomly occluded sound with random duty cycle.

Figure 11 depicts the tracking performance during the robotic trials for all three sound signals in terms of the tracking error θ_*e*_. In the robotic trials, the robot's performance is relatively good. Small errors in tracking are observed during the trials as evident from the recorded video footage. Even after undertaking compensatory actions as described in Section 3.3, errors in tracking are unavoidable under real-life conditions due to ambient noise introduced in the sound signals. The robot manages to compensate for any positive or negative tracking errors (that are introduced by respectively turning either too fast or too slow) for a given loudspeaker by respectively making relatively smaller or larger turns for the next loudspeaker. This is because the difference signal *x*_(_*t*) generated by the peripheral auditory model also provides some information regarding the sound direction (see Section 2.1) that the neural mechanism uses to automatically compensate for tracking errors, even though the synaptic weights are fixed.

The tracking errors are relatively greater for the randomly occluded sound signal as compared to those for the unoccluded and periodically occluded signals. There is a consistent offset from the reference that implies that the robot's turns consistently lag behind the currently playing loudspeaker. This is in agreement with the consistent offset between the alignment of the robotic agent and the angular location of the sound signal (i.e., a consistently non-zero tracking error) observed in simulation as evident in the inset in (Figure 8C). This is because the synaptic weights learned for a randomly occluded sound do not correspond to any single sound emission duty cycle. Instead, the algorithm learns the “best possible” values for the synaptic weights that fit all the sound emission duty cycles. This implies that in the robotic trials there will always be an offset between the angular location of the loudspeaker and the orientation of the robot as well.

In our experiments the loudspeaker sequence was never broken during a trial. Breaking the sequence, for example by not playing a given loudspeaker, the sound signal will jump forward along the trajectory by an amount that is twice the nominal displacement. For example, if the angular velocity of the sound signal is 0.5°/time step (or 5°/10 time steps) skipping a single loudspeaker would displace the sound source by 10° in 20 time steps. Assuming that the number of time steps between consecutive loudspeakers is unchanged, the angular speed will however remain unchanged. This forward jump in the sound signal will cause the synaptic weights to be updated by a relatively larger amount than usual. This would accelerate the learning during any such forward jumps in the sound signal, resulting in relatively faster convergence toward the optimal synaptic weights.

Furthermore, the loudspeaker sequence cannot be random because that would imply a sound signal moving with a randomly varying angular velocity in a randomly varying direction. For example, if the loudspeaker sequence is #1 → #2 → #7 → #4…, then the angular velocity of sound signal will vary as 0.5°/time step → 2.5°/time step → 1.5°/time step → …, and the direction of motion will vary as *left*→*left*→*right* → …. This would cause the synaptic weights to increase when the sound signal moves from left to right and decrease when it moves from right to left. The size of weight update, which corresponds to the angular speed, would vary randomly as well. As a consequence of these effects, the synaptic weights will not converge. This implies that the neural mechanism cannot learn a target angular velocity that is not constant.

We have employed a semi-circular trajectory for the sound signal in all experiments to simplify the problem of motion perception such that there is a 1:1 relationship between the agent's learned angular turning velocity and the target's angular velocity. The problem of motion perception is essentially the same in the case of a target moving along linear trajectory with a constant velocity. This is because the temporal relationship between the perceived position of a target sound signal *before* turning and *after* turning depends only on the signal's velocity and not on the shape of the trajectory. Therefore, a robotic agent using the proposed neural mechanism can still learn an angular turning velocity that corresponds to the target linear velocity. In the case of more complicated target trajectories comprising both linear and angular components, the neural mechanism may only learn the average velocity over the entire trajectory.

The neural mechanism is furthermore not limited to a specific sound frequency as its functionality is independent of the sound frequency. For a different sound frequency the peripheral auditory model generates a different difference signal that still encodes sound direction. The neural mechanism essentially uses the direction information in terms of the sign of difference signal to drive the synaptic weight updates in the right direction. However, the size of weight updates is dependent on the absolute magnitude of the difference signal. Therefore, for a different sound frequency but keeping all other neural parameters unchanged, the number of iterations taken for the synaptic weights to converge will be different.

## 5. Conclusions and future directions

We present an adaptive neural learning mechanism, derived from ICO learning, that employs a synaptic weight update rule adapted from differential Hebbian learning. The neural mechanism was able to successfully learn the constant and unknown angular velocity of a continuous unoccluded pure tone virtual sound signal moving along a semi-circular trajectory in simulation. We also investigated the performance of the neural mechanism in the presence of sparsity in acoustic stimulus. We used three different types of acoustic stimuli each having a sound frequency of 2.2 kHz–continuous unoccluded sound, periodically occluded sound with a 60% sound emission duty cycle and randomly occluded sound with random sound emission duty cycle chosen from a uniform distribution within the range 10–90%.

We first implemented an instance of the neural mechanism in simulation. The neural mechanism was able to learn the angular velocity of the continuous unoccluded sound signal in simulation for three different target angular velocities–1.5°/time step, 1.0°/time step and 0.5°/time step. We validated the acoustic tracking performance of the neural mechanism after learning via robotic trials in tracking a virtually-moving continuous unoccluded sound signal with angular velocity of 1.5°/time step.

We then investigated whether a second instance of the neural mechanism could learn the angular velocity of an target sound signal that was periodically occluded with a 60% sound emission duty cycle. The sound signal moved with a constant and unknown angular velocity along a semi-circular trajectory as before. The neural mechanism was able to learn the angular velocity of the periodically occluded sound signal in simulation for three different target angular velocities–1.5°/time step, 1.0°/time step and 0.5°/time step. We validated the acoustic tracking performance of the neural mechanism after learning via robotic trials in tracking a virtually-moving but periodically occluded sound signal with angular velocity of 1.5°/time step.

Finally we investigated whether a third instance of the neural mechanism could learn the angular velocity of a target sound signal that was randomly occluded with a randomly varying duty cycle uniformly distributed within the range 10–90%. Once again, the sound signal moved with a constant and unknown angular velocity along a semi-circular trajectory. The neural mechanism was able to learn the angular velocity of the randomly occluded sound signal in simulation for three different target angular velocities–1.5°/time step, 1.0°/time step and 0.5°/time step. We validated the acoustic tracking performance of the neural mechanism after learning via robotic trials in tracking a virtually-moving but randomly occluded sound signal with angular velocity of 1.5°/time step.

In all robotic trials the robot was relatively successful in tracking the sound signal in spite of the absence of compensation for possible detrimental effects such as ambient noise, mismatch between the robot's motor characteristics, ground friction and depletion rate of the battery.

The neural mechanism implements a purely reactive closed-loop system; the robot only turns *after* the target sound signal has moved to a new location along its trajectory and it always follows the sound signal. There is an unavoidable positive and finite delay between the target sound signal moving to its new location and the robot completing its turn. In the simulation this time delay is of one time step and in practice with the real robot it is the sum of the time step and the non-deterministic processing time in the sensorimotor loop. Predatory animals that utilise tracking behaviour, to catch prey for example, tend to be able to predict its future position. Such prediction is clearly more advantageous for the predator to minimise the neural sensorimotor delays (Nijhawan and Wu, 2009; Franklin and Wolpert, 2011) and to maximise its chances of success. Behavioural evidence for predictive tracking mechanisms has been reported in salamanders (Borghuis and Leonardo, 2015) and dragonflies (Dickinson, 2015; Mischiati et al., 2015a,b) that use vision for prey capture. In the auditory domain, the barn owl, *Tyto alba* is well known for auditory prey capture (Konishi, 1973). Both behavioural (Langemann et al., 2016) and neurophysiological (Witten et al., 2006; Weston and Fischer, 2015) evidence has been reported for auditory motion representation in the barn owl. Lizards such as the Mediterranean house geckos, *Hemidactylus tursicus*, are known to prey on crickets and have been observed to orient and navigate toward loudspeakers playing male cricket calls (Sakaluk and Belwood, 1984). However, to the best of our knowledge there is no study or evidence reported in the literature of predictive mechanisms involved in lizard acoustic prey capture. The presented neural mechanism may be used to predict the future position of the sound signal by allowing the learning to continue such that the synaptic weights increase beyond those that correspond to the actual angular velocity of the target sound signal. After successful learning the robot would then turn quickly enough to orient toward a future position of the sound signal. Such a mechanism could be considered as an internal forward model (Wolpert et al., 1995) for acoustic motion perception.

## Author contributions

DS: Scientific problem formulation, implementation, experimentation, and manuscript preparation. PM: Support in scientific discussion and manuscript preparation.

### Conflict of interest statement

The authors declare that the research was conducted in the absence of any commercial or financial relationships that could be construed as a potential conflict of interest.
