# Supplementary material for: An Adaptive Neural Mechanism for Acoustic Motion Perception with Varying Sparsity
Source: Front Neurorobot. 2017 Mar 9;11:11. doi: 10.3389/fnbot.2017.00011 (PMC5343069; doi:10.3389/fnbot.2017.00011)
Supplement: Supplementary file 4 [file Image1.PDF]

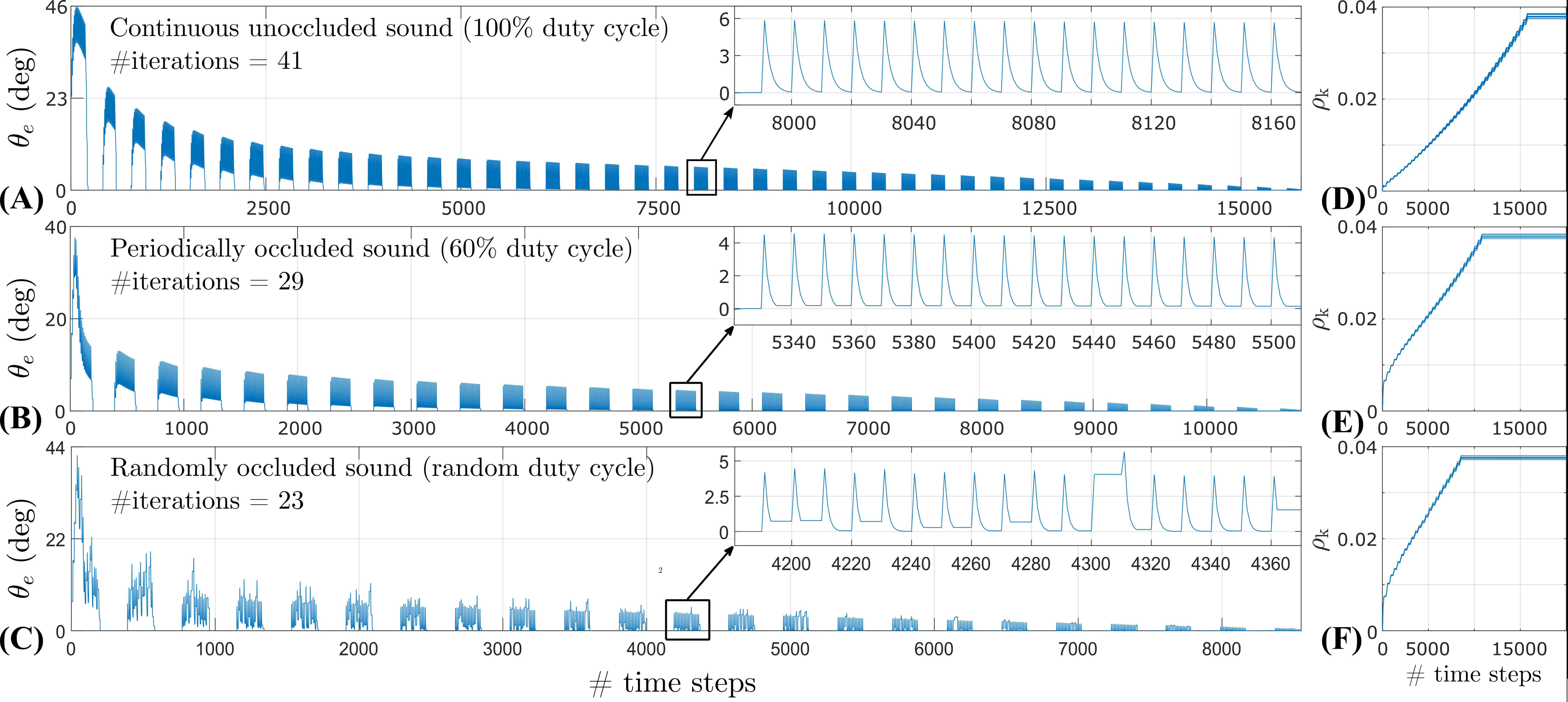

Figure 8-1. Tracking error θ_e for a target angular velocity of 1.0°/time step for varying duty cycles of sound emission. **A** Continuous unoccluded sound. **B** Periodically occluded sound with 60 % duty cycle. **C** Randomly occluded sound with random duty cycle. The insets show θ_e for a single iteration as an example. **D** Synaptic weights for continuous unoccluded sound. **E** Synaptic weights for periodically occluded sound with 60 % duty cycle. **F** Synaptic weights for randomly occluded sound with random duty cycle.
