# Supplementary material for: An Adaptive Neural Mechanism for Acoustic Motion Perception with Varying Sparsity
Source: Front Neurorobot. 2017 Mar 9;11:11. doi: 10.3389/fnbot.2017.00011 (PMC5343069; doi:10.3389/fnbot.2017.00011)
Supplement: Supplementary file 5 [file Image2.PDF]

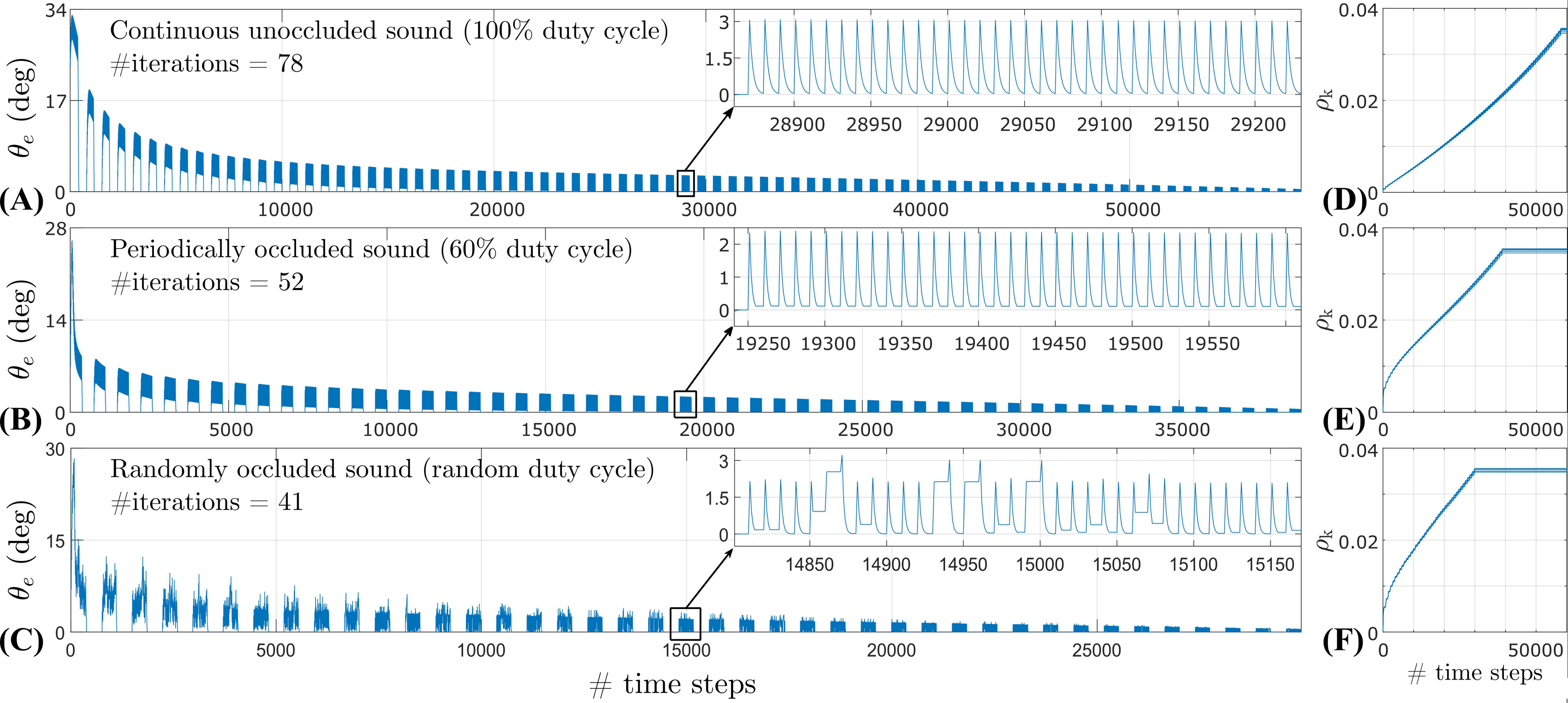


**Figure 8-2.** Tracking error $\theta_e$ for a target angular velocity of $0.5^\circ$/time step for varying duty cycles of sound emission. **A** Continuous unoccluded sound. **B** Periodically occluded sound with 60 % duty cycle. **C** Randomly occluded sound with random duty cycle. The insets show $\theta_e$ for a single iteration as an example. **D** Synaptic weights for continuous unoccluded sound. **E** Synaptic weights for periodically occluded sound with 60 % duty cycle. **F** Synaptic weights for randomly occluded sound with random duty cycle.
