# Supplementary material for: An Adaptive Neural Mechanism for Acoustic Motion Perception with Varying Sparsity
Source: Front Neurorobot. 2017 Mar 9;11:11. doi: 10.3389/fnbot.2017.00011 (PMC5343069; doi:10.3389/fnbot.2017.00011)
Supplement: Supplementary file 7 [file Image4.PDF]

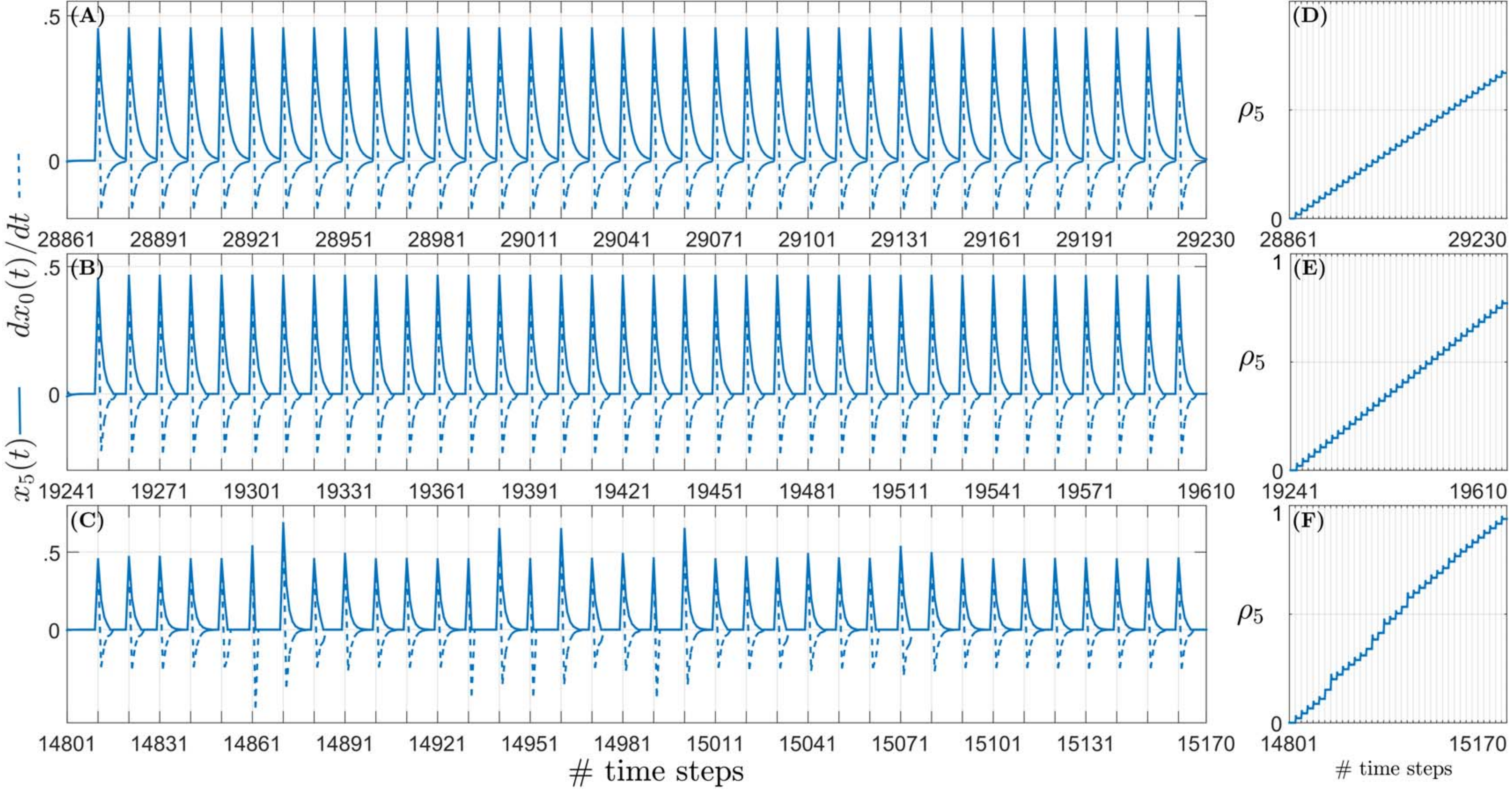

Figure 9-2. Snapshots of the synaptic weight updates (right column) corresponding to the correlation (left column) between the predictive signal $x_5(t)$ (solid line) and the derivative of retrospective signal $\frac{dx_0(t)}{dt}$ (dashed line) for a sound signal moving with an angular velocity of $0.5°$/time step. **A** Continuous unoccluded sound. **B** Periodically occluded sound with 60 % duty cycle. **C** Randomly occluded sound with random duty cycle. **D** Synaptic weights for continuous unoccluded sound. **E** Synaptic weights for periodically occluded sound with 60 % duty cycle. **F** Synaptic weights for randomly occluded sound with random duty cycle.
